# Progressive Early Image Recognition for Wireless Vision Sensor Networks

**DOI:** 10.3390/s22176348

**Published:** 2022-08-24

**Authors:** AlKhzami AlHarami, Abubakar Abubakar, Bo Zhang, Amine Bermak

**Affiliations:** 1 Division of Information and Computing Technology, College of Science and Engineering, Hamad Bin Khalifa University, Doha P.O. Box 34110, Qatar; 2 Visual Computing Group, Microsoft Research Asia, Beijing 100080, China

**Keywords:** image recognition, image reconstruction, image restoration, smart cameras

## Abstract

A wireless vision sensor network (WVSN) is built by using multiple image sensors connected wirelessly to a central server node performing video analysis, ultimately automating different tasks such as video surveillance. In such applications, a large deployment of sensors in the same way as Internet-of-Things (IoT) devices is required, leading to extreme requirements in terms of sensor cost, communication bandwidth and power consumption. To achieve the best possible trade-off, we propose in this paper a new concept that attempts to achieve image compression and early image recognition leading to lower bandwidth and smart image processing integrated at the sensing node. A WVSN implementation is proposed to save power consumption and bandwidth utilization by processing only part of the acquired image at the sensor node. A convolutional neural network is deployed at the central server node for the purpose of progressive image recognition. The proposed implementation is capable of achieving an average recognition accuracy of 88% with an average confidence probability of 83% for five subimages, while minimizing the overall power consumption at the sensor node as well as the bandwidth utilization between the sensor node and the central server node by 43% and 86%, respectively, compared to the traditional sensor node.

## 1. Introduction

In recent years, the idea of Internet-of-Things (IoT), which was once a mere ambitious thought, has become a reality. At its core, IoT is all about a worldwide network of unique and identifiable objects that communicate using standard protocols [1]. Realization of this concept has to do with embedding sensors in objects that are able to record information from their environments and transmit the information back to the central server. These sensors are now becoming smart, as they are capable of doing far more than just recording and relaying the information to the central server [2]. IoT realization has contributed largely to a significant boom in smart sensor design for various fields.

One such field that has seen significant progress is the field of image sensors. The advancements made in this field spewed out what is referred to as wireless vision sensor networks (WVSNs). The WVSN system consists of multiple vision sensor nodes (VSNs)—often a smart camera; a central server node or base station (BS); and in some instances, an aggregation node [3]. The realization of WVSNs has unlocked the potential for many applications, such as Machine Vision [4], environmental monitoring [5], surveillance [6,7,8], and wireless capsule endoscopy [9].

**Figure 1:**
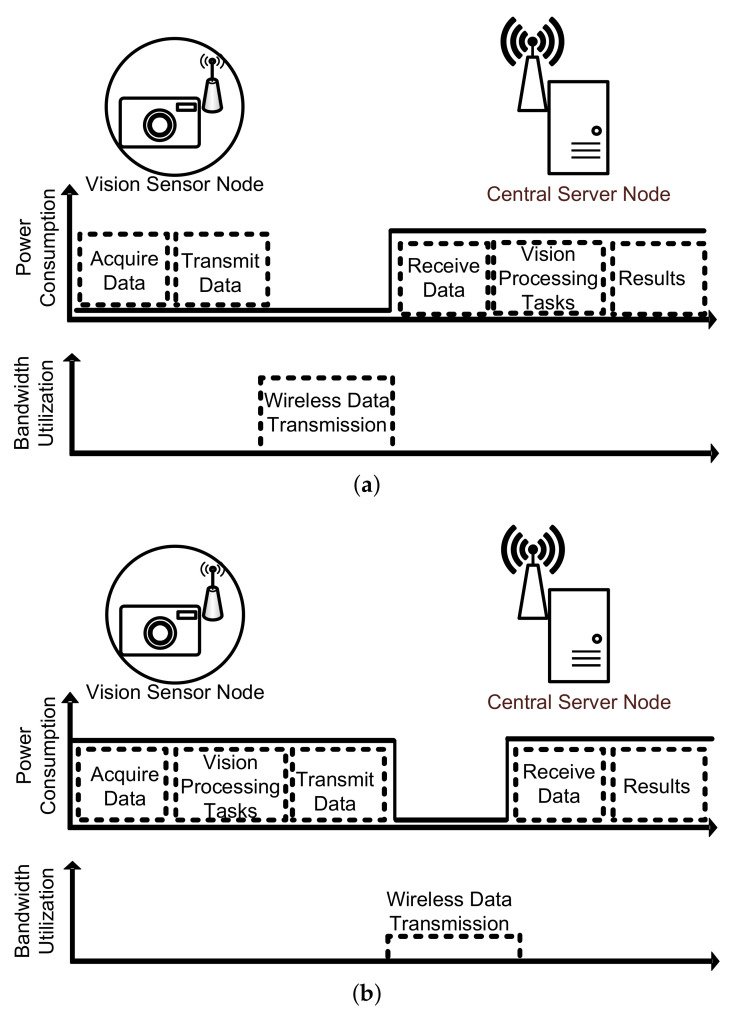
WVSN architectures. (**a**) No vision processing at sensor node; (**b**) all vision process at sensor node.

In most applications, the vision sensor node is expected to perform some vision processing tasks, in what represents a marked progression from traditional vision sensors. This, however, has added new design constraints in terms of power consumption and bandwidth, among others [10]. The design trade-off between power consumption and bandwidth is particularly dominant in WVSN realization. As the vision sensors are often placed far away from the central server node, it is necessary to minimize the amount of information that is relayed back to the server over the limited bandwidth of the wireless communication link. One of the ways to achieve this is by adding a dedicated digital signal processor (DSP) at the sensor node that can compress the captured raw visual data. While this is quite effective in minimizing the bandwidth utilization, it adds significant overhead in terms of power consumption [11]. Even state-of-the-art DSPs that use industry standard compression techniques like JPEG codes can add power consumption overhead in the same order as the vision sensor itself [12,13]. As shown in Figure 1, this key trade-off between power consumption and bandwidth is what guides any implementation of a WVSN [4,5,6,14,15].

In the architecture presented in Figure 1a, the vision sensor is not designed to handle any vision processing task. It only captures the raw visual data and transmits it wirelessly to the central server node. This strategy has the advantage of low design complexity as well as the minimal processing power expenditure at the sensor node. However, as earlier stated, the bandwidth utilization is significant as large raw data needs to be transmitted to the central server node [5,6,7,8,9,10,11,12,13,14]. Conversely, the second architecture illustrated in Figure 1b involves implementing some vision processing tasks at the VSN and transmitting only the processed information to the server [15,16]. Intuitively, it is clear that the communication energy expenditure is minimized at the expense of increased hardware complexity and processing power at the VSN.

The complicated trade-off between processing power consumption and bandwidth utilization is not trivial, and this has necessitated the emergence of a third architecture that strives to strike a balance between the two illustrated in Figure 1. This architecture involves the distribution of the vision processing tasks between the VSN and the central server node [17]. This is especially useful in machine vision applications [18] where pre-processing tasks such as segmentation can be conducted at the sensor node while the remaining tasks, often more complex, are completed at the server node. Meanwhile, implementing fewer tasks at the sensor node will save on power consumption; it was shown in [4] that there is still a need for a compression algorithm to minimize bandwidth utilization. This opened the door for many compression algorithms that are tailored for deployment at the sensor nodes of WVSN systems.

Unlike the mainstream image compression algorithms, designing compression algorithms for WVSNs requires careful design considerations [19,20]. The first consideration is the compression ratio. This factor represents the actual reduction in size of image data represented. While it is important to have a high compression ratio to save on storage and transmission bandwidth, it should not come at the cost of severe degradation in image quality. The image data received at the central server node should still be a good representation of the captured raw image data at the sensor node. Furthermore, a high compression ratio should not be achieved through complex implementation at the sensor node as that will increase power consumption at the sensor node.

Under these design guidelines, the Microshift algorithm, which is one of the most recent and effective compression algorithms for WVSNs, was proposed in [21]. This compression algorithm is very hardware friendly, in addition to yielding a high compression ratio. A key feature of this algorithm is its progressive decompression capability, which perfectly suits the architecture of WVSNs. The algorithm divides the full image into nine subimages and progressively compresses them at the sensor node. This progressive compression saves power, as the sensor node does not compress the full image at once. In a similar way, the progressive transmission of the subimages saves on bandwidth. The subimages are decompressed in the sequential order they arrive at the central server node.

Inspired by the features of the Microshift algorithm, we propose a WVSN implementation that is capable of achieving progressive image recognition. Firstly, the processing power consumption at the sensor node and transmission bandwidth are minimized by progressively compressing and transmitting five out of the nine subimages. Secondly, a convolution neural network (CNN) is deployed at the central server node to perform progressive image recognition on the subimages as they arrive. Last but not least, we present the trade-off necessary between recognition accuracy and expenditure in terms of overall power and bandwidth utilization.

The rest of this paper is organized as follows: Section 2 presents the implementation of the complete WVSN; experiment results are discussed in Section 3; while conclusions are drawn in Section 4.

## 2. Proposed Wireless Vision Sensor Network (WVSN) Implementation

**Figure 2:**
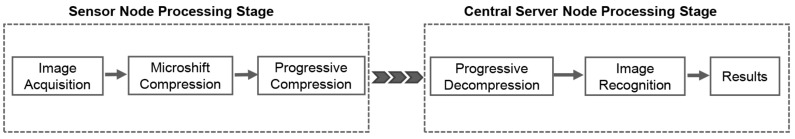
Proposed wireless vision sensor network implementation.

In this section, the details of the proposed WVSN implementation are presented. The system as a whole can be divided into two processing stages, namely the sensor node processing stage and the central server node processing stage, as illustrated in Figure 2.

### 2.1. Sensor Node Processing Stage

The processing capability of this stage is limited and, therefore, few processes are implemented. As illustrated in Figure 2, this stage commences with the VSN recording the raw image data. Regarding processing at this stage, both power consumption and bandwidth utilization are taken into account. The processing tasks at this stage are as follows.

#### 2.1.1. Microshift Compression

The Microshift compression algorithm reported in [21] is an efficient state-of-the-art image compression algorithm designed for hardware. In addition to achieving good compression performance with low power consumption, the Microshift algorithm has been shown to be viable for integration with the modern CMOS image sensor architecture. Implementation of the Microshift algorithm is summarized as follows:

- A 3×3 Microshift pattern, known to both encoder and decoder, is defined as follows:
  $$M_{shift}=[\begin{array}{ccc} \delta_{0} & \delta_{1} & \delta_{2}\\ \delta_{5} & \delta_{4} & \delta_{3}\\ \delta_{6} & \delta_{7} & \delta_{8} \end{array}]$$
  where$\delta_{i}$where di is the Microshift value for the pixel located at position ‘*i*’.
- The 3×3 Microshift pattern is replicated and applied across the whole image through pixel-wise additions, so that each pixel is micro-shifted by a particular value.
- The micro-shifted image is now quantized with a coarse quantizer of smaller resolution (e.g., 3 bit) to generate the sub-quantized micro-shifted image $\tilde{I}$:
  (1) $$\tilde{I}=Q\left(I+M\right)$$
  where Q(·) denotes quantization operation for each pixel, *I* is the captured image, and *M* is the matrix that contains replication of the Microshift pattern that covers the full captured image.

#### 2.1.2. Progressive Compression

To improve the compression ratio, the Microshift algorithm utilizes a further lossless encoding step. The implementation steps are summarized below:

- The sub-quantized micro-shifted image, $\tilde{I}$, is downsampled. This is achieved by extracting pixels having the same Microshift value. A total of nine subimages are therefore obtained. One advantage of this downsampling process is that large areas of uniform regions become apparent and subimages are therefore more compressible. Another key advantage of this is that the subimages can be progressively compressed and transmitted to the central server node and as a result, decompression can also be progressive. For low-power WVSNs, this feature is vital.
- For the progressive compression, the first subimage ${\tilde{I}}_{1}$ is first compressed using a technique termed “intra-prediction” that uses only the information in the particular subimage. Intra-prediction involves a typical lossless prediction scheme.
- For the remaining subimages, they are compressed using “inter-prediction”. This technique uses the information from the previously compressed subimage. This means that the pixels in the second subimage will be predicted based on the pixels from the first subimage, while the third subimage is predicted using information from the first and second subimages, and so on.
- This process is termed “Progressive Compression” because as soon as a subimage is compressed, the compressed bitstream is transmitted wirelessly to the central server node.

For WVSNs, this process reduces the power consumption at the vision sensor node as the full image is not compressed in its entirety at once. Furthermore, the progressive transmission of bits reduces the bandwidth demand since bits of the subimages are sent sequentially as opposed to sending the compressed bits of the full image.

### 2.2. Central Server Node Processing Stage

The available processing power available at this stage is quite large compared to the sensor node processing stage. As shown in Figure 2, the process completed at the sensor node is reversed at the central server node, in addition to other complex vision processing tasks. In the proposed WVSN, the following tasks are implemented.

#### 2.2.1. FAST Progressive Decompression

The proposed system is designed around the progressive decompression capability of WVSNs. As earlier stated, the compressed bits are transmitted from the sensor node to the central server node in the sequential order of subimages. As these compressed bits are received at the central server node, the respective subimages are reconstructed using the “FAST Decompression Algorithm” presented in [21].

The FAST Decompression algorithm merits its name from its speed of execution. It is inspired by the “Heuristic Decompression” in [22]. This technique can recover the original image I, from the sub-quantized micro-shifted image $\tilde{I}$. The technique exploits the correlation between neighboring pixels, which is evident in most natural images. The implementation involves obtaining the best estimation of the pixel by refining its uncertainty range using its neighbors in a 3×3 neighborhood patch.

Let us assume that a pixel $I_{i}$ from the original image takes the value ${\tilde{I}}_{i}$ in the sub-quantized micro-shifted image. It is worth remembering that this pixel was shifted by $\delta_{i}$ before quantization. Therefore, it is effectively quantized to ${\tilde{I}}_{i}$−$\delta_{i}$ and its uncertainty range is given by (2).

(2) $${⋓}_{i}=\left[{\tilde{I}}_{i}-\delta_{i},{\tilde{I}}_{i}-\delta_{i}+△\right]$$

where △ is the quantization step

**Figure 3:**
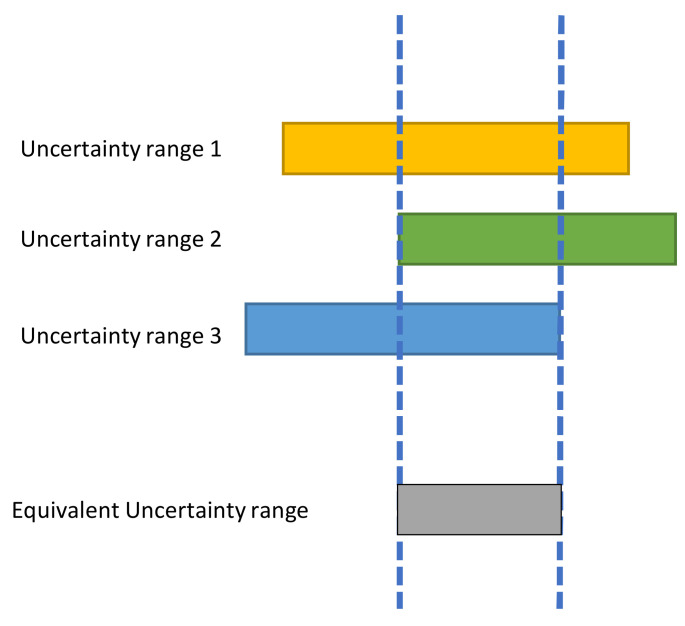
Heuristic decompression to show how the equivalent uncertainty range of a pixel is determined using two neighbors.

For each of the neighboring pixels in the patch containing ${\tilde{I}}_{i}$, their respective uncertainty range is similarly determined. The refined uncertainty range of ${\tilde{I}}_{i}$ is obtained as the intersection of its uncertainty range and that of its neighbors, as illustrated in Figure 3.

To implement the FAST decompression algorithm progressively is quite easy. For any subimage ${\tilde{I}}_{i}$ whose bits arrive at the central server node, it is reconstructed using the pixels of the subimages ${\tilde{I}}_{1}$ to ${\tilde{I}}_{i-1}$. For the pixel locations of the bits that are yet to arrive, bilinear interpolation is employed to account for them. For example, the procedure that will eventually lead to the reconstruction of the fifth subimage is as follows:

(3) $${\tilde{I}}_{1}\to {\tilde{I}}_{2}$$

(4) $$\left\{{\tilde{I}}_{1},{\tilde{I}}_{2}\right\}\to {\tilde{I}}_{3}$$

(5) $$\left\{{\tilde{I}}_{1},{\tilde{I}}_{2},{\tilde{I}}_{3}\right\}\to {\tilde{I}}_{4}$$

(6) $$\left\{{\tilde{I}}_{1},{\tilde{I}}_{2},{\tilde{I}}_{3},{\tilde{I}}_{4}\right\}\to {\tilde{I}}_{5}$$

**Figure 4:**
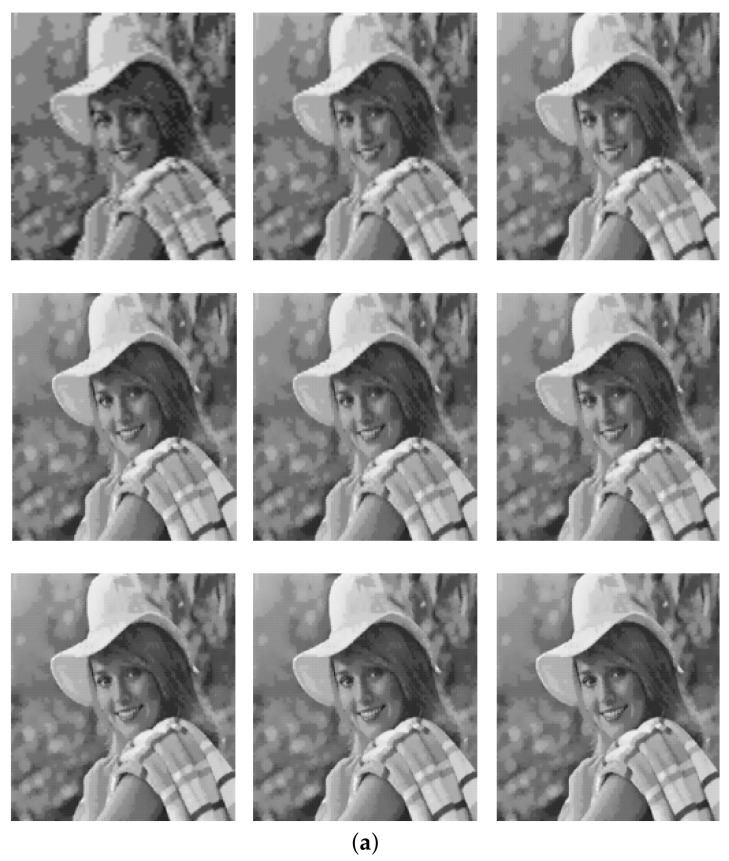

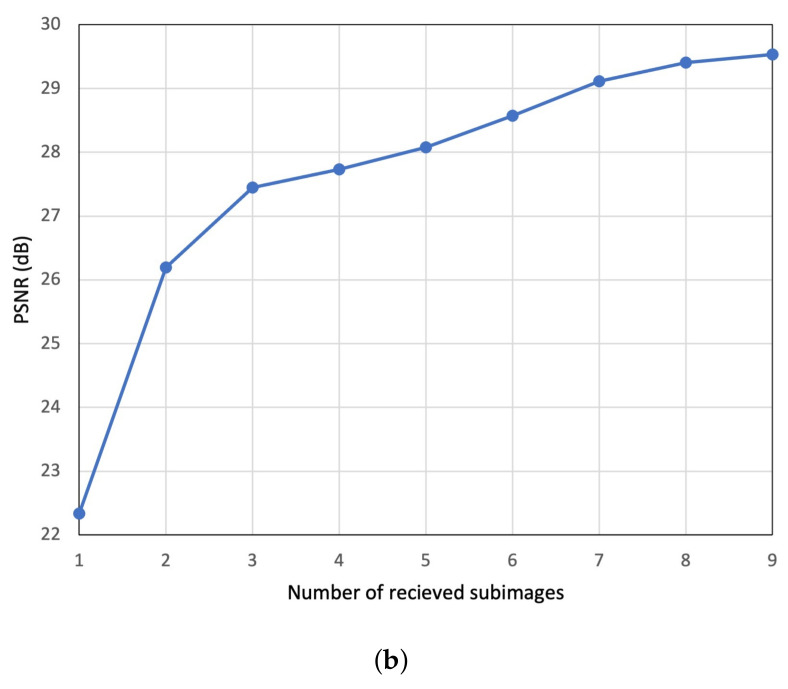
Progressive decompression of Elaine test image. (**a**) Improvement in visual quality as more subimages are received. (**b**) Trend of PSNR score as subimages are received.

The progressive decompression method provides improvement in reconstructed subimages as more bits are received. An illustration of the progressive Decompression using the “Elaine” standard test image is shown in Figure 4.

Figure 4a presents the visual results of the progressive decompression using the chosen test image. As more subimage bits reach the central server node, the visual quality of the reconstructed subimages progressively improves. The trend of the quantitative result from the progressive decompression in terms of peak signal-to-noise ratio (PSNR) value is presented in Figure 4b. It is clearly seen how the PSNR score increases as more subimages are received. Therefore, by establishing this agreement between the visual and quantitative results, the functionality of the progressive decompression is verified.

#### 2.2.2. Neural Network

A convolutional neural network (CNN) is deployed at the central server node for image recognition. CNNs are a replication of the human brain for machine learning applications and have been integral components in the history and development of deep learning [23]. Instead of training the CNN from scratch, transfer learning is applied to a pre-built and pre-trained model. A pre-trained model has already learned how to extract the important features from natural images. Often, in cases where the dataset is limited, it is useful to implement a pre-trained model that already has fixed weights for a particular application [24]. In [25], it was claimed that rather than using randomly initialized weights, a better performance may be achieved by using pre-trained weights from a distant task.

**Figure 5:**
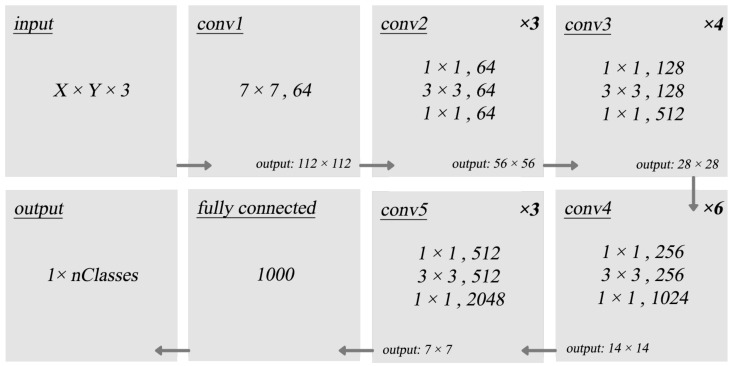
Architecture of ResNet50 base model.

There are many pre-trained CNNs available. These include GoogleNet, VGGNet, ALexNet and Resnet. In this work, the pre-trained CNN is based on the ResNet50 base model illustrated in Figure 5. The model was pre-trained on the ImageNet dataset [26] that consists of 1.2 million images. The base model consists of a series of convolution layers, skip connections, average pooling and an output fully connected (dense) layer.

The weights of the ResNet50 are frozen and the output layer is replaced with a new layer. The new output layer will be retrained on the Caltech101 [27] dataset that is tested in this work and has a 101 output number that matches the number of dataset classes. All inputs are pre-processed by resizing them to match the network input.

#### 2.2.3. Testing Dataset

The Caltech101 dataset contains around 8677 images divided randomly into 101 classes. The data is partitioned with 80% used for neural network training and 20% for testing.

The experiment was conducted on the progressively decompressed Caltech101 dataset that is generated in this work. Each image was converted into nine progressively decompressed subimages and the recognition algorithm was performed on each one.

## 3. Experimental Results and Discussions

In this section, the proposed WVSN implementation is analyzed to verify its performance in terms of power consumption and bandwidth utilization.

### 3.1. Image Recognition

**Figure 6:**
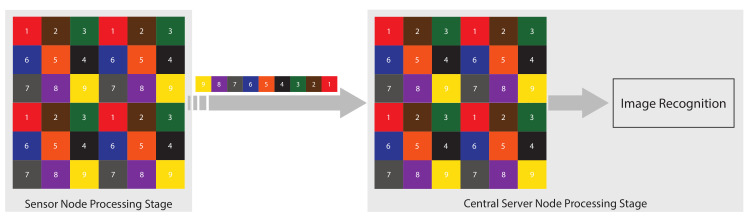
WVSN with standard image recognition.

The proposed WVSN implementation is capable of progressive image recognition. Recognition in the proposed work was completed progressively using the subimages. Typically, the decoder at the central server node waits for all the subimages to arrive, recombines them, decompresses the recombined image, and finally runs it through the recognition neural network, as illustrated in Figure 6. However, this is quite inefficient for low-power and low-bandwidth WVSNs. This is why the proposed WVSN model conducts recognition progressively on the subimages as they arrive. As the subimages are received, they are progressively decompressed and passed through the neural network (NN) for the purpose of image recognition.

In this experiment, we observed how recognition accuracy trends relative to the increase in number of received subimages. Transfer learning was employed for the purpose of training. The weights of the model’s convolutional layers were frozen or unchanged, while the output layer of the ResNet50 model was removed and replaced with a new output layer. The network was then trained on the Caltech101 testing dataset and reported an accuracy of 94.9%.

For image compression, we employed the Microshift algorithm that is capable of achieving, on average, a compression ratio of around 5.6–5.7 (approximately 1.3 bits per pixel). We simulated the central server node by using progressive decompression and recognition.

#### 3.1.1. Average Results

**Figure 7:**
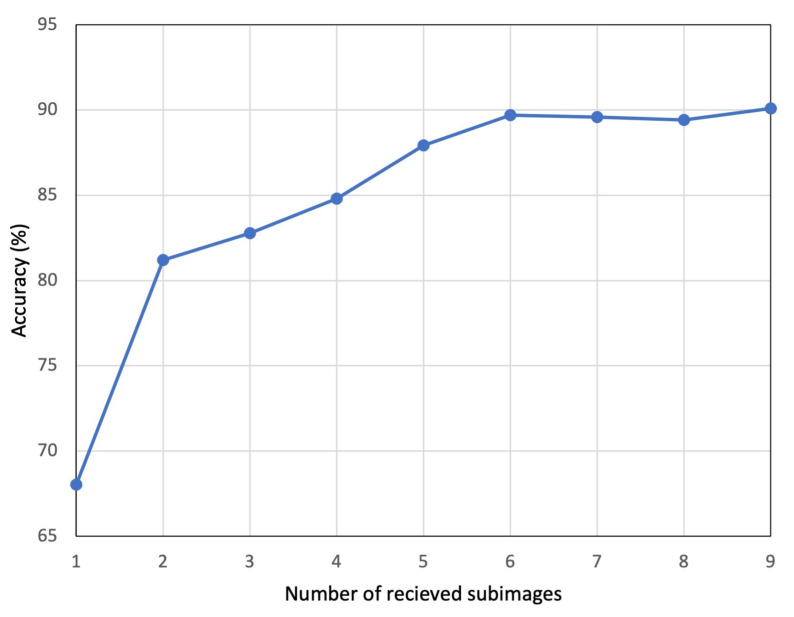
Average recognition accuracy trend with increase in number of subimages received.

The average progressive recognition accuracy for the generated testing dataset with increasing number of received subimages is presented in Figure 7.

The observed trend from Figure 7 shows an increase in recognition accuracy as more subimages are received. The lowest recognition accuracy was around 68% when the first subimage was received. The accuracy steadily increased to a maximum of around 90% when all nine subimages were received, for the available testing dataset. This steady increase was in total agreement with the results in Figure 4a,b. Intuitively, it makes sense that as more subimages arrive, the image quality was improved and consequently, recognition accuracy was enhanced.

**Figure 8:**
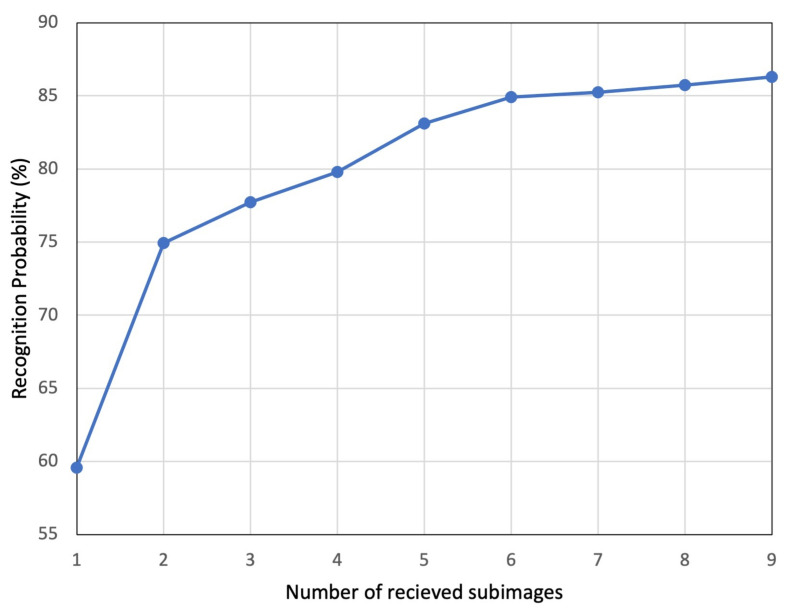
Average probability trend with increase in number of subimages received.

To further highlight the effectiveness of the progressive recognition, we present in Figure 8 the trend of average recognition probability as the number of received subimages increases. The trend of average probabilities presented was used to estimate the confidence level of the correct prediction. The trend shows a marked growing level of confidence from 60% to 86% of the proposed model to make correct predictions as more subimages are received, meaning clearer images.

#### 3.1.2. Single Image

**Figure 9:**
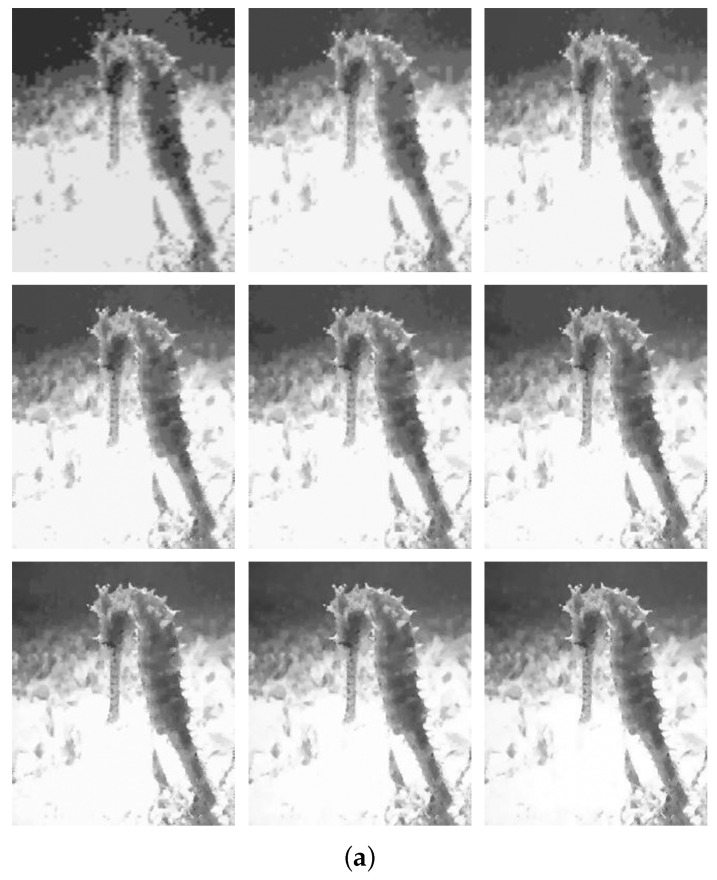

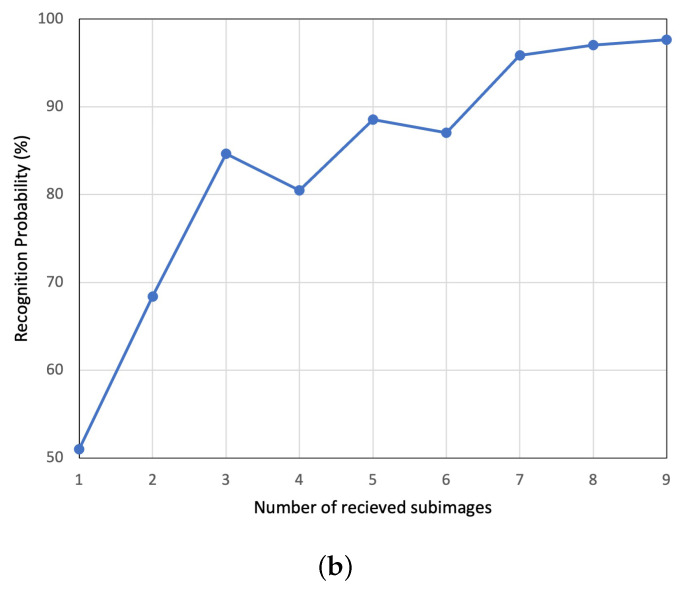
Progressive recognition of Caltech101 Seahorse test image. (**a**) Improvement in visual quality as more subimages are received. (**b**) Probability trend with increase in number of subimages received.

Figure 9a illustrates a generated progressively decompressed images of a ’seahorse’ category from the Caltech101 dataset. The NN was able to recognize the image correctly starting with the first subimage, with a probability of 51%. As it can be noticed in Figure 9b, the probability was steadily increasing to 89% when five subimages were received.

### 3.2. Performance Comparison

**Figure 10:**
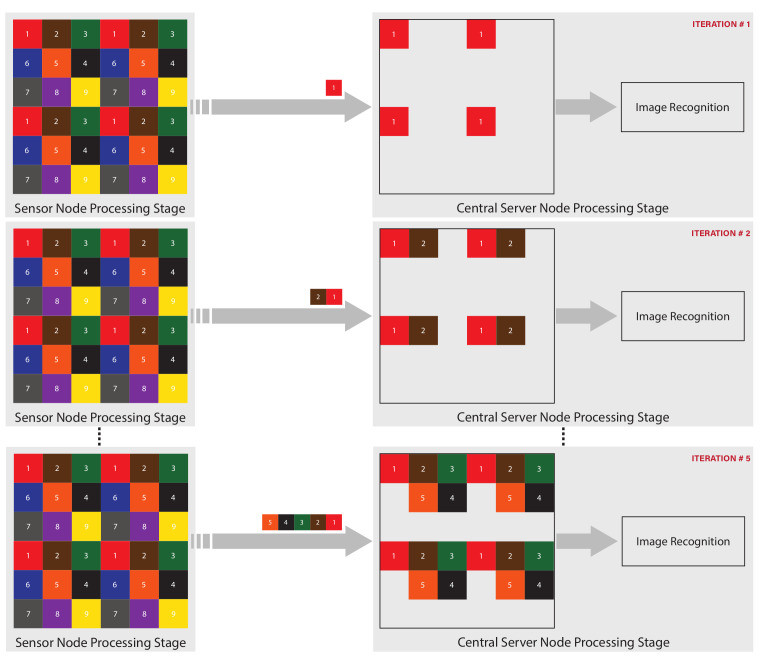
WVSN progressive image recognition.

From Figure 7 and Figure 8, it makes sense to utilize the full set of subimages for recognition to achieve a higher accuracy rate with significant confidence. However, the cost of processing and transmitting all the subimages from the sensor node to the central server node needs to be addressed. There needs to be a balance between recognition accuracy and expenditure in terms of hardware resources, power consumption and bandwidth utilization. To achieve this, in our proposed WVSN implementation, we suggest trading-off recognition accuracy. To realize the trade-off, we propose to compress and transmit five of the nine subimages, which only reduces the average accuracy and average recognition probability, respectively, by 2% and 3%. It should be stated here that the central server node is not idling, rather, it is continuously running recognition on the reconstructed image as the subimages arrive, as illustrated in Figure 10.

To validate the benefits of the implemented WVSN, a performance comparison is presented in terms of hardware resource utilization, bandwidth utilization and overall power consumption.

#### 3.2.1. Hardware Resource Utilization

In Table 1, we compare the hardware resource utilization of the WVSN when the full nine subimages are utilized with the performance when only five subimages are utilized. Both designs were synthesized using the TSMC 0.18 μm library in Synopsys Design Compiler.

From Table 1, it is evident that processing only five subimages significantly reduces the amount of hardware resources that are utilized. For example, there is a significant reduction in the number of registers used, which helps to reduce the area utilization of the compression core. There is also a reduction in the other building blocks (adders/subtractors, multiplexers and shifters), which points to a decrease in algorithm complexity at the sensor node.

#### 3.2.2. Bandwidth Utilization

Assuming a WiFi transmission channel between the sensor node and the central server node that is capable of transmitting data at 40 Mbps at 0.00525 μW/bit [28], we compare in Table 2, using the “Elaine” test image, our WVSN implementation with five subimages and the implementation with nine subimages. We also extend the comparison to cover other reported hardware algorithms, as well as the case of a traditional sensor node that simply acquires the image and transmits it to the central server node. For the reported algorithms, the total encoded bits are estimated by multiplying the average bits per pixel (bpp) with the input image size.

For the assumed WiFi channel transmitting at 40 Mbps, the time required to transmit one bit evaluates to 25 ns. From Table 2, it can be seen that there is a direct relationship between the number of bytes to be transmitted and the bandwidth utilization in terms of transmission time. This is why reducing the number of bytes to be transmitted through the use of compression algorithms is essential in any WVSN implementation. It is also observed from Table 2 that our WVSN implementation is less costly in terms of bandwidth utilization quantified by the transmission time.

#### 3.2.3. Overall Power consumption

To validate the benefits of any WVSN implementation, the overall power consumption needs to be minimized when compared to the traditional sensor node that simply acquires and transmits the image to the central server node. The overall power consumption ($P_{T,w}$) of any WVSN implementation is a cumulative sum of three factors: (1) the power consumption of the image acquisition ($P_{acq,w}$); (2) the power consumption of the image processing ($P_{pr,w}$); (3) the power consumption of the transmission of data to the central server node ($P_{tr,w}$), as given by Equation (7).

(7) $$P_{T,w}=P_{acq,w}+P_{pr,w}+P_{tr,w}$$

In the traditional sensor node, the overall power consumption ($P_{T,t}$) is determined by the acquisition power consumption ($P_{acq,t}$) and the transmission power consumption ($P_{tr,t}$), as given by Equation (8).

(8) $$P_{T,t}=P_{acq,t}+P_{tr,t}$$

It should be stated that the acquisition power consumption is similar for both our WVSN implementation and the traditional sensor node, i.e., $P_{acq,w}=P_{acq,t}$. This is because our WVSN implementation works on the Capture↦Store↦Compress↦Transmit paradigm. Comparing Equations (7) and (8), it can be said that for our WVSN implementation to be worthwhile, it needs to satisfy the condition:

(9) $$P_{tr,w}+P_{pr,w}<P_{tr,t}$$

For our WVSN implementation that needs to send a total of 7681 bytes (Table 1) across the WiFi channel at 0.00525 μW/bit, the transmission power ($P_{tr,w}$) is determined to be 0.323 mW; while $P_{tr,t}$ evaluates to 2.32 mW for a total of 55,296 bytes. From Table 1, the processing power consumption of our WVSN implementation obtained from the Synopsys Design Compiler was 1.0036 mW. Therefore, the respective overall power consumption of our proposed WVSN implementation, as well as the traditional sensor node, are calculated as:

(10) $$P_{T,w}=P_{acq,w}+1.33\;\mathrm{mW}$$

(11) $$P_{T,t}=P_{acq,t}+2.32\;\mathrm{mW}$$

In Equation (10) and (11), it is seen that our WVSN implementation significantly reduces the overall power by around 43%. It is also obvious that the transmission power is quite dominant in the determination of the overall power consumption of WVSN implementations. Therefore, while trying to maintain an acceptable recognition accuracy and probability, we opted to utilize five out of nine subimages in order to reduce the transmission power.

#### 3.2.4. ASIC Implementation

**Figure 11:**
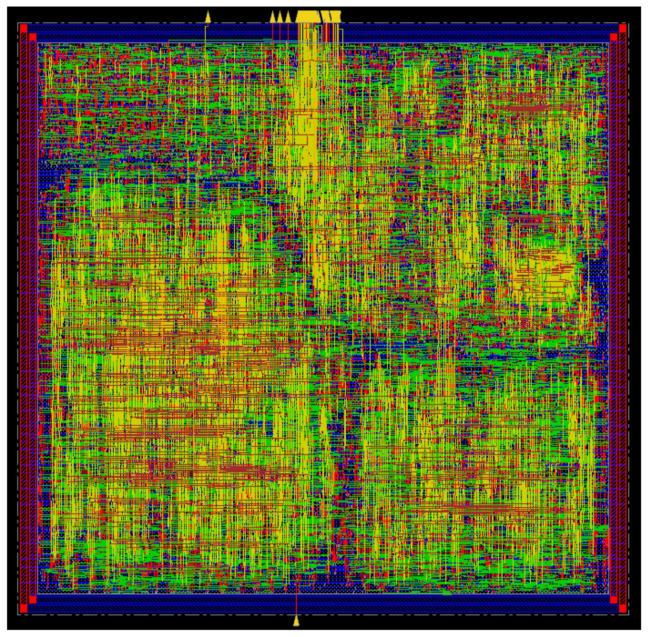
Layout of the implemented WVSN sensor node.

For our WVSN implementation, the full design flow was accomplished. First, the design was completed in Verilog, generated by MATLAB HDL Coder, then synthesized using the TSMC 0.18 μm standard library in Synopsys Design Compiler, and finally a Layout was generated using Cadence Innovus Software. The layout of the implemented WVSN sensor node is presented in Figure 11.

## 4. Conclusions

In this paper, we have proposed a wireless vision sensor network implementation that is capable of early and progressive image recognition. The proposed implementation works on the subimages transmitted by the sensor node and progressively runs them through a recognition algorithm, as they are decompressed at the central server node. While maintaining a high level of accuracy with significant confidence level, the progressive processing of images helps to save power and transmission bandwidth. In the implemented WVSN, 2% of the average recognition accuracy was traded-off to minimize expenditure in terms of both sensor node overall power as well as bandwidth utilization, respectively, by 43% and 86%. As part of future work, the implemented WVSN could still be improved by training the neural network model on images with lower quality as well as using a compression algorithm that yields an even greater compression ratio. Furthermore, in the implemented WVSN, a fixed number of subimages is used, however, that can be improved by having a handshaking protocol between the sensor node and the central server node. This will allow the central server node to instruct the sensor node to stop the transmission of subimages when an acceptable recognition probability is achieved.

## Figures and Tables

**Table 1:** Comparison of hardware resource utilization between the proposed WVSN with five subimages and nine subimages.

Resource	WVSN with 9 Subimages	Proposed WVSN with 5 Subimages
Image Size	256 × 216	256 × 216
Bytes to be transmitted	9823	**7681**
Compression ratio	5.6294	**7.1991**
Adders/Subtractors	173	**117**
Total 1 Bit Registers	8641	**1580**
Multiplexers	525	**362**
Shifters	47	**16**
Power Consumption	1.6543 mW	**1.0036 mW**

**Table 2:** Bandwidth utilization comparison of WVSN implementations.

	Method				
Parameter	Traditional Sensor Node	PSD [29]	Block-Based [30]	Compressive Sensing [31]	Proposed WVSN (5 Subimages)
Image Size	256 × 216	256 × 216	256 × 216	256 × 216	256 × 216
Bytes to be transmitted	55,296	20,736	14,515	42,854	**7681**
Transmission time (ms)	11.06	4.15	2.90	8.57	**1.54**

## Data Availability

The data supporting the reported results were conducted by the authors and are available on request from the corresponding author.

## References

[1] Kokkonis G., Psannis K.E., Roumeliotis M., Schonfeld D. (2016). Real-time wireless multisensory smart surveillance with 3D-HEVC streams for internet-of-things (IoT). J. Supercomput..

[2] Iqbal M.A., Bayoumi D.M. (2019). Wireless sensors integration into internet of things and the security primitives. Int. J. Comput. Netw. Commun. IJCNC.

[3] Tsai T., Huang C., Chang C., Hussain M.A. (2020). Design of Wireless Vision Sensor Network for Smart Home. IEEE Access.

[4] Imran M., Khursheed K., O’Nils M., Lawal N. Exploration of target architecture for a wireless camera based sensor node. Proceedings of the NORCHIP 2010.

[5] Ferrigno L., Marano S., Paciello V., Pietrosanto A. Balancing computational and transmission power consumption in wireless image sensor networks. Proceedings of the IEEE Symposium on Virtual Environments, Human-Computer Interfaces and Measurement Systems.

[6] Gasparini L., Manduchi R., Gottardi M., Petri D. (2011). An Ultralow-Power Wireless Camera Node: Development and Performance Analysis. IEEE Trans. Instrum. Meas..

[7] Bakkali M., Carmona-Galán R., Rodriguez-Vazquez A. A prototype node for wireless vision sensor network applications development. Proceedings of the 2010 5th International Symposium On I/V Communications and Mobile Network.

[8] Kerhet A., Magno M., Leonardi F., Boni A., Benini L. (2007). A low-power wireless video sensor node for distributed object detection. J. -Real-Time Image Process..

[9] Deligiannis N., Verbist F., Iossifides A.C., Slowack J., Van de Walle R., Schelkens P., Munteanu A. (2012). Wyner-Ziv video coding for wireless lightweight multimedia applications. Eurasip J. Wirel. Commun. Netw..

[10] Olyaei A., Genov R. Mixed-signal CMOS wavelet compression imager architecture. Proceedings of the 48th Midwest Symposium on Circuits and Systems.

[11] Karlsson J. (2010). Wireless Video Sensor Network and Its Applications in Digital Zoo. Ph.D. Thesis.

[12] Woo J., Sohn J., Kim H., Yoo H. (2009). A 152-mW Mobile Multimedia SoC With Fully Programmable 3-D Graphics and MPEG4/H.264/JPEG. IEEE Trans. Very Large Scale Integr. Syst..

[13] Tang F., Chen D.G., Wang B., Bermak A. (2013). Low-Power CMOS Image Sensor Based on Column-Parallel Single-Slope/SAR Quantization Scheme. IEEE Trans. Electron Devices.

[14] Soro S., Heinzelman W.B. (2009). A Survey of Visual Sensor Networks. Adv. Multim..

[15] Kandhalu A., Rowe A., Rajkumar R. DSPcam: A camera sensor system for surveillance networks. Proceedings of the 2009 Third ACM/IEEE International Conference on Distributed Smart Cameras (ICDSC).

[16] Rowe A., Goode A.G., Goel D., Nourbakhsh I. (2007). CMUcam3: An Open Programmable Embedded Vision Sensor.

[17] Bailey D.G. (2011). Design for Embedded Image Processing on FPGAs.

[18] Imran M., Khursheed K., Lawal N., O’Nils M., Ahmad N. (2012). Implementation of Wireless Vision Sensor Node for Characterization of Particles in Fluids. IEEE Trans. Circuits Syst. Video Technol..

[19] Mammeri A., Hadjou B., Khoumsi A. (2012). A Survey of Image Compression Algorithms for Visual Sensor Networks. Int. Sch. Res. Not..

[20] Kaddachi M.L., Soudani A., Lecuire V., Torki K., Makkaoui L., Moureaux J.M. (2012). Low power hardware-based image compression solution for wireless camera sensor networks. Comput. Stand. Interfaces.

[21] Zhang B., Sander P.V., Tsui C., Bermak A. (2019). Microshift: An Efficient Image Compression Algorithm for Hardware. IEEE Trans. Circuits Syst. Video Technol..

[22] Wan P., Au O.C., Pang J., Tang K., Ma R. High bit-precision image acquisition and reconstruction by planned sensor distortion. Proceedings of the 2014 IEEE International Conference on Image Processing (ICIP).

[23] Goodfellow I., Bengio Y., Courville A. (2016). Deep Learning.

[24] Islam M.M., Tasnim N., Baek J.H. (2020). Human Gender Classification Using Transfer Learning via Pareto Frontier CNN Networks. Inventions.

[25] Yosinski J., Clune J., Bengio Y., Lipson H., Ghahramani Z., Welling M., Cortes C., Lawrence N.D., Weinberger K.Q. (2014). How transferable are features in deep neural networks?. Advances in Neural Information Processing Systems 27.

[26] Deng J., Dong W., Socher R., Li L.-J., Li K., Fei-Fei L. Imagenet: A large-scale hierarchical image database. Proceedings of the IEEE Conference on Computer Vision and Pattern Recognition.

[27] Li F.-F., Andreeto M., Ranzato M.A., Perona P. (2022). Caltech 101 (Version 1.0). CaltechDATA. https://data.caltech.edu/records/20086.

[28] Minoli D. (2013). Building the Internet of Things with IPv6 and MIPv6.

[29] Howard P.G., Vitter J.S. Fast and efficient lossless image compression. Proceedings of the DCC ’93: Data Compression Conference.

[30] Zhang M., Bermak A. (2010). Compressive Acquisition CMOS Image Sensor: From the Algorithm to Hardware Implementation. IEEE Trans. Very Large Scale Integr. Syst..

[31] Dadkhah M., Jamal Deen M., Shirani S. (2014). Block-Based CS in a CMOS Image Sensor. IEEE Sensors J..

